# Moxibustion for cephalic version: a feasibility randomised controlled trial

**DOI:** 10.1186/1472-6882-11-81

**Published:** 2011-09-26

**Authors:** Carole K Do, Caroline A Smith, Hannah Dahlen, Andrew Bisits, Virginia Schmied

**Affiliations:** 1 School of Biomedical and Health Sciences, University of Western Sydney, Locked Bag 1797, Penrith South DC, NSW 2751, Australia; 2 Centre for Complementary Medicine Research, University of Western Sydney, Locked Bag 1797, Penrith South DC, NSW 2751, Australia; 3 Family and Community Health Group, School of Nursing and Midwifery, University of Western Sydney, Locked Bag 1797, Penrith South DC, NSW 2751, Australia; 4 Royal Women's Hospital, Sydney, Australia; 5 School of Nursing and Midwifery, University of Western Sydney, Locked Bag 1797, Penrith South DC, NSW 2751, Australia

**Keywords:** breech, moxibustion, randomised controlled trial, feasibility

## Abstract

**Background:**

Moxibustion (a type of Chinese medicine which involves burning a herb close to the skin) has been used to correct a breech presentation. Evidence of effectiveness and safety from systematic reviews is encouraging although significant heterogeneity has been found among trials. We assessed the feasibility of conducting a randomised controlled trial of moxibustion plus usual care compared with usual care to promote cephalic version in women with a breech presentation, and examined the views of women and health care providers towards implementing a trial within an Australian context.

**Methods:**

The study was undertaken at a public hospital in Newcastle, New South Wales, Australia. Women at 34-36.5 weeks of gestation with a singleton breech presentation (confirmed by ultrasound), were randomised to moxibustion plus usual care or usual care alone. The intervention was administered over 10 days. Clinical outcomes included cephalic presentation at birth, the need for ECV, mode of birth; perinatal morbidity and mortality, and maternal complications. Feasibility outcomes included: recruitment rate, acceptability, compliance and a sample size for a future study. Interviews were conducted with 19 midwives and obstetricians to examine the acceptability of moxibustion, and views on the trial.

**Results:**

Twenty women were randomised to the trial. Fifty one percent of women approached accepted randomisation to the trial. A trend towards an increase in cephalic version at delivery (RR 5.0; 95% CI 0.7-35.5) was found for women receiving moxibustion compared with usual care. There was also a trend towards greater success with version following ECV. Two babies were admitted to the neonatal unit from the moxibustion group. Compliance with the moxibustion protocol was acceptable with no reported side effects. Clinicians expressed the need for research to establish the safety and efficacy of moxibustion, and support for the intervention was given to increase women's choices, and explore opportunities to normalise birth. The sample size for a future trial is estimated to be 381 women.

**Conclusion:**

Our findings should be interpreted with caution as the study was underpowered to detect statistical differences between groups. Acceptance by women and health professionals towards moxibustion suggest further research is warranted.

**Trial Registration:**

Australia and New Zealand Clinical Trials Register (ANZCTR): ACTRN12609000985280

## Background

Three to four percent of babies at full term are in a breech presentation [1]. To reduce the incidence of babies in a breech presentation external cephalic version (ECV) and postural management have been used to correct the presentation prior to term. Whilst ECV at term has been shown to be successful [2] ECV between 32-34 weeks is not successful and there is insufficient data regarding effectiveness of ECV between 34 and 36 weeks [3]. Recently reported findings from the Early ECV trial at 24-35 weeks versus 37 or more weeks reported an increased likelihood of cephalic presentation at birth but no reduction in the rates of caesarean delivery, or in the risk of preterm birth [4]. ECV may not be acceptable to all women [5], and some women express a preference for a vaginal birth [6-8], therefore there remains a need to examine the effectiveness and safety of other options of care.

Traditional Chinese medicine (TCM) has a tradition of using moxibustion for postural management. This method dates back about 1300 years [9] and is commonly used in primary healthcare systems in East Asia [10]. Moxibustion is a technique which generates heat by burning a herbal preparation containing *Artemisia vulgaris *(mug-wort), applied close to the skin until it produces hyperaemia from local vasodilatation. To promote cephalic version, moxibustion is applied to the acupuncture point bladder 67 (BL67 (located on outer corner of the fifth toenail)). Treatment regimes vary with no consensus on the best regime, however applying moxibustion for 15-20 minutes, from one to ten times daily up to 10 days is common in clinical practice [11]. The general mechanism of moxibustion is proposed to be a combination of thermal (infrared radiation) and pharmacological action of the materials used [12,13]. Moxibustion sticks have been shown to emit primarily long-wavelength infrared radiation (IR-C) indicating that moxibustion mainly affects the superficial skin, where heat receptors are located [13]. Due to the limited skin penetration of IR-C moxibustion sticks thermal effects on internal organs are more likely due to arise from reflex mechanisms [14].

Evidence of the clinical effectiveness of moxibustion has been summarised in two systematic reviews [15,16]. In the most recent review an increased rate of cephalic version was found in the moxibustion group compared to the control group (Relative Risk (RR), 1.36, 95% confidence interval, 1.17-1.58) [15]. There were no significant differences in any adverse clinical outcomes between groups, although numbers were small. Whilst these results were encouraging, there was significant heterogeneity between trials, and further high quality research is needed. Evaluating unfamiliar interventions from different cultural contexts require preliminary evaluation through feasibility studies. However, limited studies of moxibustion have been successfully implemented for breech presentation in Western maternity settings, and the influence of cultural unacceptability resulting in poor compliance and early stopping was evident in at least one trial of moxibustion [17]. Recently two studies reported positive experiences from women on using moxibustion, with high compliance, and few problems [5,18]. However the views of health care providers identify a need for further research to demonstrate clinical and cost effectiveness [5]. We report on the findings from a feasibility study of moxibustion compared with usual care in a randomised controlled trial (RCT) for women with a breech presentation. We also examined the acceptability of implementing a trial within an Australian context among pregnant women and their health care providers.

## Methods

This study was conducted at the John Hunter Hospital, a public hospital in Australia. Human ethics approval was obtained from the Hunter New England Human Research Ethics Committee of Hunter New England Health (EC00403) to conduct the clinical trial and interviews with health professionals. Women were eligible if they were aged greater than 18 years, at 34-36.5 weeks of gestation with a singleton breech presentation (confirmed by ultrasound), and normal fetal biometry. Early intervention at 34-36 weeks was planned prior to the fetus descending into the pelvis, to allow sufficient time for evaluation of moxibustion prior to the timing of the ECV. Exclusion criteria included: twin pregnancy, risk of premature birth, heart or kidney diseases affecting the mother, placenta previa, history of ante-partum haemorrhage, intrauterine growth restriction, hypertensive disease, isoimmunisation, previous uterine operations, uterine anomaly, pre-labour rupture of the membranes, multiple pregnancy, fetal congenital abnormality, contraindication to vaginal delivery and fetal death in utero.

Randomisation was computer generated, and concealed in opaque sealed envelopes. Women were allocated to a study group moxibustion plus usual care or usual care alone by the research midwife taking the next sequentially numbered envelope.

The treatment protocol was developed by CS a qualified acupuncturist with 15 years experience of treating women with acupuncture during pregnancy. The protocol was based on the review of interventions reported in a systematic review [15], and Debra Betts an experienced acupuncture clinician (personal communication September 2009). Women allocated to moxibustion were requested to attend (with their partner/support person if available) for a training and demonstration session by the research midwife. This involved advice and demonstration on how to use the moxibustion safely, and instructions on its use at home. The woman or her partner/support person was trained to light the moxibustion sticks and to hold an individual stick one thumb width above the acupuncture point BL67 (located on the lateral side of the 5th toe) over individual feet consecutively. Smokeless and odourless moxibustion sticks were used. Advice on the intensity of stimulation was given advising the participant not to let the points become uncomfortably hot. Participants were requested to perform this treatment for 10 minutes on each foot with a total of 20 minutes per treatment, twice a day (one in the morning and one in the evening at a time suitable for the participant) for ten days. The participant was given a ten-day supply of moxibustion sticks. Women allocated to the control group received usual antenatal care. Antenatal care for women during this period involved weekly visits with midwives and obstetricians as per hospital protocols. No additional care was made to women with a breech presentation for example on postural support, although individual clinicians may provide advice to women. All participants were required to return to the research midwife 10 days following randomisation for monitoring. A repeat ultrasound was undertaken to confirm the position of the baby for all participants. If the follow up ultrasound confirmed a breech presentation women had the option for an ECV.

### Data collection and analysis

Clinical outcomes included cephalic presentation at birth, the need for ECV, mode of birth, perinatal morbidity and mortality, maternal complications, and adverse events. We collected data on the number of eligible women with a breech presentation at 34-36 weeks, willingness of participants to be randomised, women's views on participation in the trial, response rates to questionnaires, compliance with the intervention and aimed to estimate the effect size for a fully powered trial. The research midwife collected baseline data on clinical, demographic and socio-economic characteristics. Women completed a self report questionnaire after each moxibustion session on fetal movement, and uterine contractions. Following completion of the intervention participants were asked to complete questions on the acceptability of the trial.

### Sample size and analysis

This was a feasibility study, and it was estimated that a sample of 30 women would be sufficient to provide data to answer our study questions. Clinical outcome data were analysed using 'an intention to treat' approach, and the initial analysis examined the demographic and baseline characteristics of women randomised to the trial. For differences between groups testing of categorical data was undertaken using Fishers exact tests and T tests for continuous data, relative risks and 95% confidence intervals were reported. Levels of significance were reported at p < 0.05. Descriptive statistics were used to assess the feasibility questions. Analysis was performed blind to study group by a study statistician.

### Interviews with health professionals

To examine the views of health professionals towards the acceptability of moxibustion, and views on the RCT we interviewed participants. We planned to undertake a minimum of 15 interviews, using a purposive and snow-ball sample of health professionals to achieve opinions from both the midwives and doctors. Flyers were distributed in the antenatal clinics and personal approaches made to clinical staff to recruit participants to the interviews. The interviews were undertaken by CD a junior research assistant. The interviews typically explored the participant's views towards moxibustion, complementary and alternative medicine (CAM) and the feasibility study. Each interview was digitally recorded. Interviews were transcribed verbatim by CD and checked for transcription accuracy. Each transcript was read separately and independently by CD and CS. Data was subject to a process of coding and thematic analysis based on notions of consistency, commonality and the function and effects for thematic analysis [19]. The research approach was mostly inductive where the common patterns came from the data. The data was subjected to coding after repeated readings of the whole transcript to stay close to the data and maintain the context. To begin with data was subject to open coding to identify first order concepts, and then axial coding to develop categories. Categories were developed including: keeping birth normal, increasing women's choices, establishing the evidence base, and the need for safety and effectiveness.

## Results

**Figure 1:**
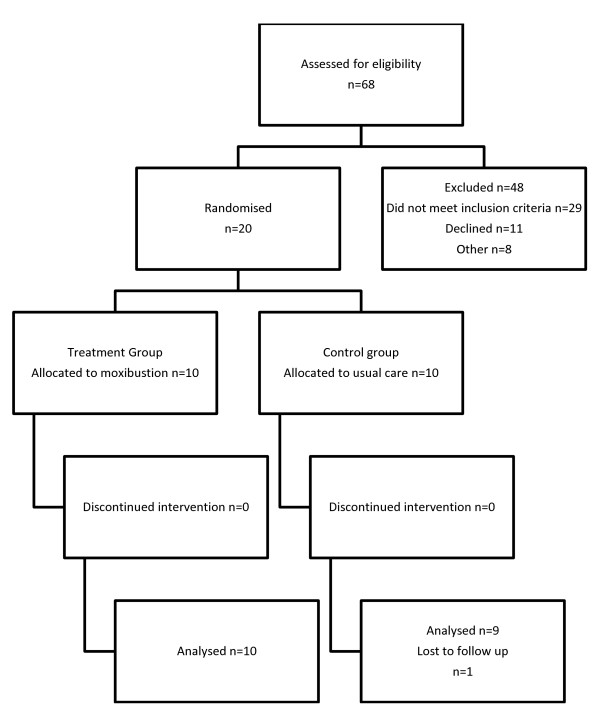
**Flow of participants through the trial**.

Between December 2009 and June 2010, 68 women with a breech presentation were approached to participate in the study between, 57% met the entry criteria. Twenty nine women did not meet the entry criteria. This included 13 women confirmed with a cephalic presentation, nine with a gestational age of greater than 36.3, three not booked with the hospital, and four with medical conditions. Eleven women declined to participate in the study, including two too busy, five refused randomisation, two had pre booked ECV or elective caesarean section, and two were not interested in the intervention. Recruitment was slower than anticipated and was stopped once 20 women had been randomised (Figure 1).

### Outcome data

Clinical outcome data was available for all women. Baseline characteristics of study participants are presented in Table 1, and were mostly similar between groups. Most women were Caucasian, nulliparous, employed, had completed tertiary education, and were at a gestational age of 35 weeks.

**Table 1:** Baseline characteristics of women at trial entry

	Moxibustion		Control	
	N = 10	%	N = 10	%
Age (years)	30.36	(±3.13)	24.60	(±5.23)
Parity 0	7	70.0	9	90.0
Marital status: Married/defacto	9	90.0	8	80.0
Employment status				
Working	6	60.0	4	40.0
Unemployed	1	10.0	2	20.0
Home duties/student	3	30.0	4	40.0
Education*				
Completed High school	1	11.1	0	0.0
Completed tertiary education	8	88.9	8*	88.9
Ethnicity				
Caucasian	10	100.0	9	90.0
Asian	0	0.0	1	10.0
Gestational age (weeks)	34.8	(±0.69)	35.6	(±0.70)
Type of breech				
Complete	1	10.0	2	20.0
Frank	3	30.0	3	30.0
Unknown	6	60.0	5	50.0
Engagement of presenting part				
Yes	1	10.0	0	0.0
No	9	90.0	10	100.0
Placental location				
Anterior	5	50.0	1	10.0
Posterior	4	40.0	6	60.0
Other	1	10.0	3	30.0

* missing data Data are n and % or mean and standard deviation.

Analyses on clinical outcomes are presented in Table 2. Five infants in the moxibustion group were cephalic at delivery (includes before ECV and following successful ECV), compared with one in usual care only (RR 5.0, 95% confidence interval (CI) 0.70-35.5, p = 0.11). Two women in the moxibustion group had cephalic version from ECV compared with no women in usual care only group (RR 5.0, 95% CI 0.27-95.62, p = 0.28). The position of the placenta at trial entry was examined for women with cephalic presentation and four women with cephalic presentation had a placenta in a posterior position. Six women in the moxibustion group had a caesarean delivery compared with nine in usual care (RR, 0.67, 95% CI 0.39-1.15, p = 0.15). There were three cases of preterm birth in the moxibustion group compared with nil in the usual care group. None of these infants had Apgar scores < 7 at 5 minutes, and two infants from the moxibustion group were admitted to intensive care. One infant was admitted to intensive care for two days for respiratory support and one infant for seven days with intrauterine growth restriction and a possible fetal anomaly.

**Table 2:** Comparison of Outcome Measures Between Moxibustion and Control

	Moxibustion (as referent)	Control	Relative Risk (95% CI)	P value
	N = 10	N = 10		
Cephalic presentation (at delivery or before ECV)	2 (20.0)	0 (0)	5.0 (0.27-92.62	0.28
Cephalic presentation at delivery (including successful ECV)	5 (50.0)	1 (10.0)	5.0 (0.70-35.5)	0.11
ECV				
Attempted	4 (40.0)	6 (60.0)	0.67 (0.27-1.66)	0.38
Successful*	2 (20.0)	0 (0.0)	5.0 (0.27-95.62)	0.28
Mode of delivery				
Vaginal	4 (40.0)	1 (10.0)	4.0 (0.54-29.8)	0.18
Caesarean	6 (60.0)	9 (90.0)	0.67 (0.39-1.15)	0.15
Other outcomes				
Preterm birth requiring hospitalisation	2 (20.0)	0 (0)	5.0 (0.27-92.62)	0.28
Prelabour rupture of membranes at < 37 weeks	3 (30.0)	0 (0)	7.0 (0.41, 120.16)	0.18
Gestational age at delivery	38.7 (2.11)	39.9 (3.68)		0.43
Apgar score < 7 at 5 min	0 (0)	0 (0)	N/A	
Birthweight (g)	3224 (653.9)	3193 (674)		0.92
Admission to NICU	2 (20.0)	0 (0)	N/A	

Data are n (%) or mean (standard deviation) unless otherwise specified.* Calculated among women attempting ECV

Caesarean delivery was selected as the primary outcome for a sample size calculation for a future trial. Due to small numbers from this study a more conservative estimate 10% effect size was made. To be able to detect a 10% reduction in caesarean section rate for breech presentation with an alpha of 0.05 powered at 80% 173 women per arm would be required in a two arm trial. To allow for a 10% loss or withdrawal from the study, the trial will require 381 participants. Based on 15% of babies at term are in a breech position, and a recruitment rate of 29% a multi-centre site of 4-5 centres would be needed.

### Acceptability of the intervention

Ten women in the moxibustion group and one woman in the usual care group completed the questionnaire describing women's views on the acceptability of the trial. Eight women in the moxibustion group and nine women in the control said they would agree to participate in the study again if presented with this option. One women in the moxibustion group reported she was more anxious about her health and her baby' health. Women receiving moxibustion found it acceptable. No women described moxibustion as painful and there were no reported side-effects, although two women reported that it was 'hard to judge'. Three women found difficulties with administering the moxibustion, and would have appreciated additional guidance. However compliance with the treatment protocol was good with 93% compliance with twice daily administration. One woman commented she was too busy to comply with the requested schedule. Fetal movements were collected daily by women in the moxibustion group only and were reported as more than usual by 5 women, 3 women reported fetal movements as usual and 2 reported no other specific changes. There were few reports of uterine contractions following treatment, with one woman per session reporting some contractions or strong contractions.

### Analysis of interviews with health professionals

Nineteen health professionals agreed to participate in the interviews (one midwife agreed to participate at a later date but was unable to be contacted). Three participants were consultant obstetricians, one was a general practitioner, and 15 were midwives. Six were aged 36-45 years and 10 aged in the 46-55 years. Participants reported an awareness to or personal use of CAM. Ten midwives had personally used CAM. CAM modalities most frequently used included acupuncture, osteopathy, chiropractic, supplements, herbs, and massage. On the other hand, no doctors reported previous CAM use. Thirteen participants were unfamiliar with any literature on the clinical application of moxibustion for cephalic presentation.

Eighteen participants expressed positive views towards the use of CAM in general, and described a place for CAM in mainstream health care. The following participant's comments reflect their belief systems, perceived gaps in clinical effectiveness, and a responsibility to ensure new treatments are safe and effective.

I think it's fantastic, I would love to see eastern and western philosophies much more combined. I really obviously like traditional medicine, um, and it would be great if western medicine was more accepting of traditional medicine, in a really safe way obviously. (midwife)

I think we should access it more often. Let's move away from the pharmacological medicine, we are interfering too much. (midwife)

Others expressed more neutral views towards CAM which reflected a lack of awareness rather than an opinion unsupportive of CAM.

Um, I was probably initially quite um, sceptical, only because I've never seen it done probably, so it's more of a bias because I've never seen it. (midwife)

Participants' also spoke of CAM being beneficial to mainstream medicine and in the treatment of pregnancy related conditions.

Generally it's a favourable one because sometimes women get very frustrated with orthodox medicine and to deal with the discomforts of pregnancy so uh, I'm generally favourable towards complementary medicine because at least it represents some step that they could take to alleviate whatever is their problem. Or to deal with whatever challenges they are facing in the pregnancy. (doctor)

The views expressed towards moxibustion are reported under three main themes; need for evidence based research, increasing women's choices and normalising birth.

### The need for evidence based research

All doctors and midwives emphasised the need for evidence based research to support the future practice of moxibustion as an adjunct to usual care. Some participants expressed a view that they could not suggest the use of moxibustion or any other CAM without such research.

I think it's an adjunct and it has probably a lot of potentials and possibilities and this trial is part of a very useful way of ascertaining that. (midwife)

Other participants were positive towards moxibustion and CAM, although they expressed caution and described the need for safe practice.

Probably my biggest view would be that its evaluated and researched so that its evidence based, um in particularly if it's a medication therapy or even with moxibustion that its evidence based that shows that its not harmful, that there's nothing harmful that we're doing. (midwife)

The one concern I suppose is complacency surrounding uh the safety of such medicines generally because the substances are known relatively inert substances. So that's my only concern. Uh, as far as effective goes, I sometimes get a perception that the claims of effectiveness are overstated and so particularly when something is starting to become more common, and I know the evidence is limited, I'm very keen to see the evidence is assured so that um, we don't hand down ineffective things. (doctor)

Participants expressed a common view that moxibustion should be used with caution in pregnancy. They stressed in the absence of safety data, moxibustion and other complementary therapies could cause harm to the mother and baby if not used properly, and women needed to be aware of the side effects with regard to dosing.

You just need to make sure that it's alright and doesn't affect the baby, that's the only thing. (midwife)

I just think that with any kind of medicine it needs to be done with caution and to make sure that it's right for the individual person, what works for one person might not work for another person in the same way. So I just think it's really important that women understand that just because its complementary medicine, it doesn't mean that it still can't be damaging. They need to be very aware that you know, there are possible side-effects with that as well. (midwife)

Safe practice was also discussed in relation to their own professional practice. Clinicians described they did not know enough about moxibustion and CAM, and acknowledged that they were not qualified to recommend any form of CAM use to their patients, and doing so was outside of their scope of practice.

We're not allowed, we're not qualified really to advise on anything really, we just point women in the area of the research, and that is that, um unless we're an aromatherapist or a fully qualified a reflexologist etc etc. We can talk to women about natural remedies and complementary therapies in a broad text but we're really not permitted to talk about specifics. (midwife)

### Increasing women's choices

Midwives viewed moxibustion as having the potential to provide increased choices for women with breech presentation. Participants recalled the experience of stress and helplessness among women presenting with a breech presentation, and recalled women would try anything they could to achieve a normal birth. They felt that women should look at all options and moxibustion was a reasonable one to try as it was non-invasive and easy to use.

I guess giving them an opportunity to have another choice again of the possibility of turning this baby with moxibustion rather than an ECV or whatever, and again, you know its all about choice and I'm all for giving these women choices, lots of them, so that ultimately they can make a decision they can be happy with. (midwife)

### Normalising birth

Increasing women's choices was closely related to the theme of normalising birth. Participation in the trial was perceived to offer women a potential benefit of having a vaginal birth. Women were thought to be accepting of the study as it enabled them to have the feeling of being in control with their pregnancy, giving them hope and maintaining positive thinking.

I think it's a very reasonable option to offer women, um and I believe that they should look at all options to get the outcome that they want. Ideally being a healthy experience and a normal birth. Um, and if complementary medicine can help them in assisting/achieving that then I think its reasonable for them to have that as one of their options. (midwife)

Although moxibustion was discussed as a possible intervention to normalise birth there was some concern about raising the topic of breech presentation with pregnant women at a time when they would not usually think about it. According to hospital protocols women were told that breech presentation was normal before 36 weeks and they would not be scanned for breech presentation before 36 weeks.

...at 34 weeks if we have a breech we are still telling people that its still quite normal and then all of a sudden you're initiating an intervention so your actually, your actions are saying is that it is abnormal and we need to treat it, when in actual fact you know its not the message that we'd like to give. (midwife)

### Evaluating moxibustion in a clinical trial

Participants thought recruitment would be challenging due to the narrow window to recruit, administer the intervention and potential ECV if required and infrequent visits to the hospital at this stage of their pregnancy. There was also concern that detecting a breech presentation before 36 weeks was not a requirement in clinical practice and a loss of skill in this area may lead to missed breech presentations.

*The very small window of opportunity being two weeks. So if missed on one visit then the woman is usually another 1-2 weeks before she's seen so there's just so limited opportunities. (midwife)*.

## Discussion

Our study provides data to warrant further evaluation of moxibustion in an Australian population to assist with cephalic version. Clinical outcomes identify trends in cephalic version at birth and reduced caesarean delivery, however numbers were small with wide confidence intervals. The study demonstrated acceptability by woman with moxibustion, randomisation and participation in the trial, and compliance with completing questionnaires. Compliance with the moxibustion was acceptable but highlighted the importance to provide follow up advice for women in the first few days following the start of the intervention. Recruitment was slower than planned, with barriers to recruitment included the low prevalence of breech at 34 plus weeks, recruitment at one hospital site, and limited resources to recruit during a small window with midwifery care being undertaken in the community. Clinicians were accepting and supportive of this modality within an evidence based framework, and highlighted clinicians respect for the wishes of many women to be provided with increased choices, and for options to achieve a normal birth. However clinician support was moderated by concerns about the safety of moxibustion to the woman and her baby.

Many women use complementary therapies during pregnancy [20], although there are no accurate prevalence data on the use of moxibustion. Evidence of effectiveness remains inconclusive, and clinical trials in western settings have yet to replicate the positive results from trials in China. The most recent trial undertaken in Switzerland found no difference in version between groups [18], although these findings may have been influenced by an inadequate dose of moxibustion (one session per day of moxibustion). Our results suggest self administration of moxibustion for 20 minutes, twice a day was an acceptable dose and concur with findings by Vas et al (2009) [15], who found the intervention was acceptable to women, and was performed with high compliance. Both trials used smokeless moxa sticks, which avoided both potential toxicity from the smoke and dislike of the odour. The high compliance seen in our trial may have also been influenced by the support participants received from the research midwife.

The effects of moxibustion on fetal movements have been reported in other studies, and appear related to the administration of moxibustion. Establishing the safety of moxibustion is paramount and although no differences in adverse outcomes were found between groups, there were three cases of prelabour rupture of membranes in the moxibustion group. Preterm birth and premature rupture of membranes have not been widely reported in other trials, and in the meta-analysis of data from 383 women there were no significant differences in the rate of premature rupture of membranes in the moxibustion group (n = 5) versus control (n = 12) [15]. Future trials may wish to establish a safety monitoring committee to monitor this outcome.

There is little research exploring the clinician's perspective of moxibustion. Our findings were similar to the small study from the United Kingdom [5]. Both studies highlight the need for further research, and identified a need to address practical issues with recruiting women at the optimal gestation with sufficient time to make use of all treatment options. Our study also demonstrated stakeholders held positive views towards moxibustion and CAM in general, particularly among midwives. The motivation underlying positive views towards CAM has been proposed in several studies to be due to an affinity between the philosophies and principles of CAM and midwifery, and supporting women's choice and autonomy [21]. Findings from the clinician interviews may not represent the views of all clinicians at the host institution or be considered representative of clinicians elsewhere, however our findings indicate a supportive research culture for this topic of study.

The study has several strengths. Firstly, the trial was randomised thereby reducing selection bias. Secondly, although there is no consensus to the acceptable method of administering moxibustion for cephalic version our study dose suggests our intervention was in the therapeutic range. However, the study does have a number of limitations. Results should be interpreted with caution due to the small size. It is possible women who declined randomisation had different characteristics and outcomes to those randomised and this could be explored in future research. The self administration of moxibustion may lead to variability in the results and a future study will need to consider how variability could be reduced. Findings from the interviews maybe limited particularly from the small group of doctors interviewed, although sufficient data from midwives provides an understanding of the support and acceptability of moxibustion. Although the aim of the interviews was to capture a wide range of views, the purposive and snowball sampling approach used to identify clinicians may have resulted in some clinicians being less likely to be included [22]. In this case, selection bias may have occurred during the identification of participants to favour those with positive views to moxibustion, CAM or the RCT. Finally, reflexivity or research bias may have played a role in shaping the results. The research assistant had come from a CAM background and biased views may have unknowingly been projected during the interview process. This may have caused participants to express their views in a different way then they normally would.

The findings will influence future decisions concerning resourcing and planning for a future trial. We consider randomisation is acceptable, we will explore if the window for eligibility could be extended, an appropriate sample size has been determined allowing for attrition, the trial identified additional and ongoing in-service training to facilitate recruitment by health professionals and a need to provide additional attention to women in the intervention group. There is also a need to include a safety monitoring committee.

## Conclusion

The use of moxibustion administered over a ten day period has provided promising preliminary results to justify further research, and design feature to enhance implementation in a future study have been identified. An appropriately powered multi-centred study examining the treatment and cost effectiveness is now planned.

## Abbreviations

ECV: external cephalic version; TCM: traditional Chinese medicine; IR-C: infrared radiation; RR: relative risk, RCT: randomised controlled trial; BL67: Bladder 67; CAM: complementary and alternative medicine; CI: confidence interval.

## Competing interests

The authors declare that they have no competing interests.

## Authors' contributions

CD conducted the study including data collection, entry and analysis of interviews. CAS conceived the study, supervised CD and drafted the manuscript. HD, VS and AB contributed to the design and contributed to the interpreted to the interpretation of results, and commented on the draft of the paper. All authors read and approved the final manuscript.

## Pre-publication history

The pre-publication history for this paper can be accessed here:

http://www.biomedcentral.com/1472-6882/11/81/prepub
